# PigVar: a database of pig variations and positive selection signatures

**DOI:** 10.1093/database/bax048

**Published:** 2017-06-13

**Authors:** Zhong-Yin Zhou, Aimin Li, Newton O. Otecko, Yan-Hu Liu, David M. Irwin, Lu Wang, Adeniyi C. Adeola, Junying Zhang, Hai-Bing Xie, Ya-Ping Zhang

**Affiliations:** 1 State Key Laboratory of Genetic Resources and Evolution, and Yunnan Laboratory of Molecular Biology of Domestic Animals, Kunming Institute of Zoology, Chinese Academy of Sciences, Kunming 650223, China; 2 School of Computer Science and Engineering, Xi'an University of Technology, Xi'an, Shaanxi 710048, China; 3 Department of Molecular and Cell Biology, School of Life Sciences, University of Science and Technology of China, Hefei 230026, China; 4 Kunming College of Life Science, University of Chinese Academy of Sciences, Kunming 650204, China; 5 Laboratory for Conservation and Utilization of Bioresource, Yunnan University, Kunming 650091, China; 6 Department of Laboratory Medicine and Pathobiology, University of Toronto, Toronto, Canada; 7 School of Computer Science and Technology, Xidian University, Xi'an, Shaanxi 710071, China

## Abstract

Pigs are excellent large-animal models for medical research and a promising organ donor source for transplant patients. Next-generation sequencing technology has yielded a dramatic increase in the volume of genomic data for pigs. However, the limited amount of variation data provided by dbSNP, and non-congruent criteria used for calling variation, present considerable hindrances to the utility of this data. We used a uniform pipeline, based on GATK, to identify non-redundant, high-quality, whole-genome SNPs from 280 pigs and 6 outgroup species. A total of 64.6 million SNPs were identified in 280 pigs and 36.8 million in the outgroups. We then used LUMPY to identify a total of 7 236 813 structural variations (SVs) in 211 pigs. Positively selected loci were identified through five statistical tests of different evolutionary attributes of the SNPs. Combining the non-redundant variations and the evolutionary selective scores, we built the first pig-specific variation database, PigVar (http://www.ibiomedical.net/pigvar/), which is a web-based open-access resource. PigVar collects parameters of the variations including summary lists of the locations of the variations within protein-coding and long intergenic non-coding RNA (lincRNA) genes, whether the SNPs are synonymous or non-synonymous, their ancestral and derived states, geographic sampling locations, as well as breed information. The PigVar database will be kept operational and updated to facilitate medical research using the pig as model and agricultural research including pig breeding.

**Database URL**: http://www.ibiomedical.net/pigvar/

## Introduction

The pig is an important domestic animal and is an emerging model for medical research (1–4). In particular, the pig is considered a promising source of donor organs for transplant patients, with many studies evaluating the potential clinical application of porcine organs (5, 6). Natural and artificial selection have shaped genetic adaptations in pigs for their various environmental conditions, and thus this species displays considerable phenotypic diversity. For instance, Tibetan pigs have adaptations for life at high altitudes, and landrace pigs have been selected for lean growth (7, 8). To investigate the genetic basis of the diversity of pigs, several genetic markers, such as microsatellite and single nucleotide polymorphism (SNP), have been instrumental in research on artificial selection (7, 9), identification of quantitative trait locus (QTL) (10–12) and genome-wide association studies (GWAS) (12). SNPs are powerful markers for genetic research, especially selection analysis (7, 8, 13, 14). Structural variations are another important class of molecular markers that are associated with dramatic phenotypic changes during the domestication of pigs (7, 15). With the accumulation of SNP and structural variation data in pigs, the development of a comprehensive user-friendly database that brings together SNP and structural variation data sets and documents their selection scores would aid in the advancement of pig genomic research. Currently, the most widely used database for pig SNPs is dbSNP (Build 145: ftp://ftp.ncbi.nlm.nih.gov/snp/; last accessed January 18, 2017), which contains ∼60 M porcine SNPs. However, the SNP list in dbSNP lacks sample information on individuals, which limits the ability to conduct population genetic analyses based on these SNPs. With the development of high-throughput sequencing techniques, genome sequence data from hundreds of pigs, representing various breeds, has been generated (8, 13, 14). However, a huge amount of storage and computational resources is required to generate SNP and structural variation calls from this enormous amount of data. In addition, the different studies examining pig genomes have employed different criteria for calling variations making it difficult to combine variation from different sources.

Analysis of the patterns of SNP in one or more populations has identified many regions of the pig genome that have experienced positive selection (7, 16, 17). For example, *MC1R*, a locus with major effects on black coat color has undergone positive selection in parallel in both Asian and European domestic pigs (16, 17), while *NR6A1*, which controls number of vertebrae, has experienced strong artificial selection in European domestic pigs (7). Furthermore, structural variants at the *KIT* locus have been associated with white spotting in pigs (7). Many statistical methods, such as Tajima's D (18) and XP-CLR (19), have been developed to detect genome-wide selective signatures based on SNP data from populations. The recent increase in the amount of pig genomic data, coupled with sound statistical approaches, provides a platform for a comprehensive and easy-to-use database that focuses on variations (including SNPs and structural variations) called from next-generation sequencing (NGS) genomes and the identification of positive selection in pigs. This database should provide the needed impetus to advance pig genomic research and clinical applications.

Here, we developed a database, named PigVar, which is the first public web-based database containing high density pig whole-genome wide variation data and positive selection calls using genome data from three broad pig populations: Chinese domestic pigs, Tibetan pigs and European domestic pigs. We collected SNPs from 287 pigs, and 6 related species, published in several studies (8, 13, 14, 20, 21) or generated by our laboratory. These samples originated mainly from Eurasia and represent both domestication locations of the pig (22). Structural variations were also identified. The potential of using the pig being as a medical model is also illustrated using these SNP datasets. We employed five statistical methods to calculate genome-wide selective signals based on the SNP datasets. With PigVar, users can search for SNP and structural variation information for specific individual samples. We also annotated the variations to integrate protein and lincRNA information in addition to the annotations for the different selective signals.

## Database structure and content

**Figure 1.**
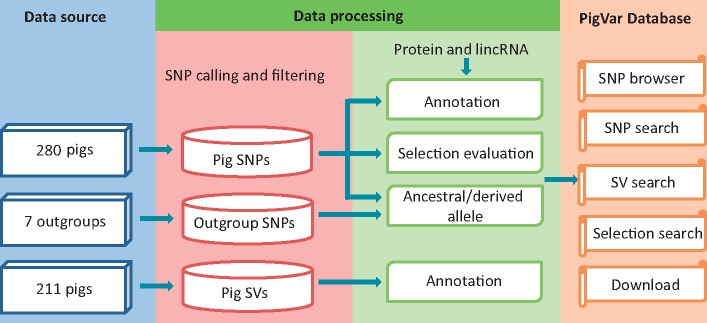
Data sources and analysis pipeline used to construct PigVar.

The PigVar database includes SNPs, structural variations and positive selection information for pigs. Geographic information for each sample is provided. Figure 1 displays the pipeline used to construct the database. A detailed description of the database is provided in the following sections.

### Data sources

We integrated genome sequence data from four published pig resequencing datasets (8, 13, 14, 20, 21) and data generated by our own group on the Diannan small ear pigs that comprised 100 Asian domestic pigs, 77 Asian wild boars (including 50 Tibetan pigs), 77 European domestic pigs, 17 European wild boars, 9 Yucatan miniature pig from Mexico and 7 individuals from 6 outgroup species (including *Sus scrofa* (Sumatra), *Sus barbatus*, *Sus verrucosus*, *Sus cebifrons*, *Sus celebensis* and *Phacochoerus africanus*). The samples have wide geographic origins, and include all locations where pigs were domesticated in Eurasia (see Supplementary Tables S1 and S2 for the complete list of individuals) (22).

### SNP calling

All genome resequencing data used here were generated using the Illumina platform. Data from Ai *et al.* (14), Li *et al.* (8) and the Korean group (20, 21) were obtained from the Sequence Read Archive (SRA). Illumina short read data downloaded from SRA and the Diannan small ear pigs (in house) were aligned using *BWA-mem*(v0.7.12-r1039) with the parameters ‘*-M -t 5’* to the pig reference genome (*Sus scrofa* 10.2) (13, 23). For data generated by Wageningen University (13), mapping files (bam files) were downloaded from the European Nucleotide Archive (ENA) and realigned to the pig reference genome by *BWA- aln*(v0.7.5a) with the parameter ‘-b’ (23). Picard (v1.119, http://broadinstitute.github.io/picard/; last accessed January 18, 2017) with the parameters ‘*REMOVE_DUPLICATES = true VALI DA T I ON  _STRINGENCY = SILENT  MAX _FILE _HANDL ES _ FOR _READ_ENDS_MAP = 1000*’ was then used to remove PCR duplicates. To obtain high-quality SNPs, *samtools mpileup* (v0.1.18, parameters: *-q 20 -Q 20 -C 50*) and the Genome Analysis Toolkit (GATK, v2.5-2-gf57256b) (24, 25) was used for SNP calling for each sample (GATK with the parameter ‘*-T RealignerTargetCreator*’), following a standard processing pipeline that included realignment of the reads around indels (GATK with the parameter ‘*-T Indel Re aligner*’) and recalibration of the base quality scores to obtain more accurate quality scores for each base(GATK with the parameter ‘*-T BaseRecalibrator*’), followed by *UnifiedGenotyper* (GATK with the parameters ‘*-T Unified Genotyper –genotype_likelihoods_model BOTH -mbq 20 -stand_call_conf 10 -stand_emit_conf 10 -L 9*’) to call SNPs. We identified at total of 64 582 787 and 36 810 479 SNPs in the pigs and their outgroup species, respectively. The number of SNPs that we identified is greater than the number found in the latest version of dbSNP (60.4 Million, Build 145: ftp://ftp.ncbi.nlm.nih.gov/snp/; last accessed January 18, 2017).

Beagle software was used to phase the SNPs identified in pigs (beagle with the parameters ‘phase-its = 40 impute-its = 10’). SNPs were then annotated with protein-coding genes, transcripts and proteins, downloaded from the Ensembl FTP site (ftp://ftp.ensembl.org/pub/release-80/gtf/sus_scrofa; last accessed January 18, 2017). In our previous study(26), which also used the *Sus scrofa* 10.2 genome, we identify 6,621 transcripts in 4,515 lincRNA genes, thus, we additionally mapped SNPs to lincRNA exons or introns.

### Ancestral and derived states

The direction (ancestral and derived) of the allelic change for each SNP was deduced by comparing the bases in 280 pigs versus 7 outgroup individuals from 6 species closely related to pigs. All outgroup individuals were considered for determining the direction of the SNP change. A SNP needed to have been sequenced in at least three of the seven outgroup individuals, and only those unambiguously called were classified. A total of 39 767 799 SNPs were classified into their ancestral and derived states from the 64.6 million pig SNPs.

### Identification of structural variation

Several structural variations are causally linked with pig domestication or adaptation to local environments (7, 14). To facilitate research on structural genomic variations in the pig, mapping files were used to identify structural variants using the LUMPY (v0.2.12) software with default parameters resulting in the identification of 7 236 813 structural variants (DEL: Deletion, INV: Inversion, DUP: Duplication, BND: Breakpoint end) in 211 pigs (samples from Ai *et al.* (14), Li *et al.* (8), the Korean group (20, 21) and our Diannan small ear pigs) (27). Data on 69 pigs generated by the Wageningen University group [11] could not be analyzed by LUMPY. Of the variants, 15 512 were annotated to protein-coding genes.

### Medical model for human diseases and traits

The pig is an excellent large animal model for medical research. To illustrate the use of our database in using pigs as medical model, we converted coordinates of SNPs in the NHGRI-EBI catalogue (http://www.ebi.ac.uk/gwas/; last accessed January 18, 2017) that are associated with human diseases and traits to the mouse and pig genomes using LiftOver (https://genome.ucsc.edu/cgi-bin/hgLiftOver; last accessed January 18, 2017), and found that the number of SNPs identified in disease and trait genes in pig is more than that found in mouse (Figure 4A). 14 060 SNPs related with human diseases and traits have homologous SNPs in pigs and are mainly associated with post bronchodilator FEV1/FVC ratio, obesity-related traits and body mass index. These results indicate that SNPs in the PigVar database should enhance research with pigs as a model for studying new treatments for human diseases.

### Selection evaluation

PigVar provides signatures of selection for Chinese domestic pigs, Tibetan pigs and European domestic pigs. Selection signals were evaluated using nucleotide diversity, Tajima’s D (18), Cockerham and Weir Fst (28), XP-EHH (29) and XP-CLR (19) (Table 1). To facilitate the identification of true signals of positive selection, users can use a set of score cutoffs that have been frequently used in recent genome-wide selection studies.

**Table 1.** Statistical terms for positive selection in the PigVar database

Statistical term	Abbreviation	Population 1	Population 2
Nucleotide diversity	PI	Chinese domestic pig	–
		European domestic pig	–
		Tibetan pig	–
Tajima's D	TD	Chinese domestic pig	–
		European domestic pig	–
		Tibetan pig	–
Cockerham & Weir FST	FST FST_POP1_POP2	Chinese domestic pig	Chinese wild boar
		European domestic pig	European wild boar
		Tibetan pig	Chinese domestic pig
Cross-population composite likelihood ratio	XP-CLR XP_CLR_POP1_POP2	Chinese domestic pig	Chinese wild boar
		European domestic pig	European wild boar
		Tibetan pig	Chinese domestic pig
Cross-population extended haplotype homozygosity	XP-EHH XP_EHH_POP1_POP2	Chinese domestic pig	Chinese wild boar
		European domestic pig	European wild boar
		Tibetan pig	Chinese domestic pig

## Database implementation

High-quality SNPs, structural variations, as well as their annotations and selection scores, were processed with Python scripts and put into MySQL (5.1.66) database (http://www.mysql.com). We use Java Server Pages (JSP, Java v1.6) to implement data visualization, search and download. Gbrowse (v2.55) was used for chromosome-based data visualization (30).

## Usage

PigVar uses a series of user-friendly interfaces to show results. In order to facilitate accessibility to the database, we provide a downloadable user guide for the database. Briefly, PigVar has five main functionalities: data retrieval, browsing, SNP search, structural variations search and positive selection search.

**Figure 2.**
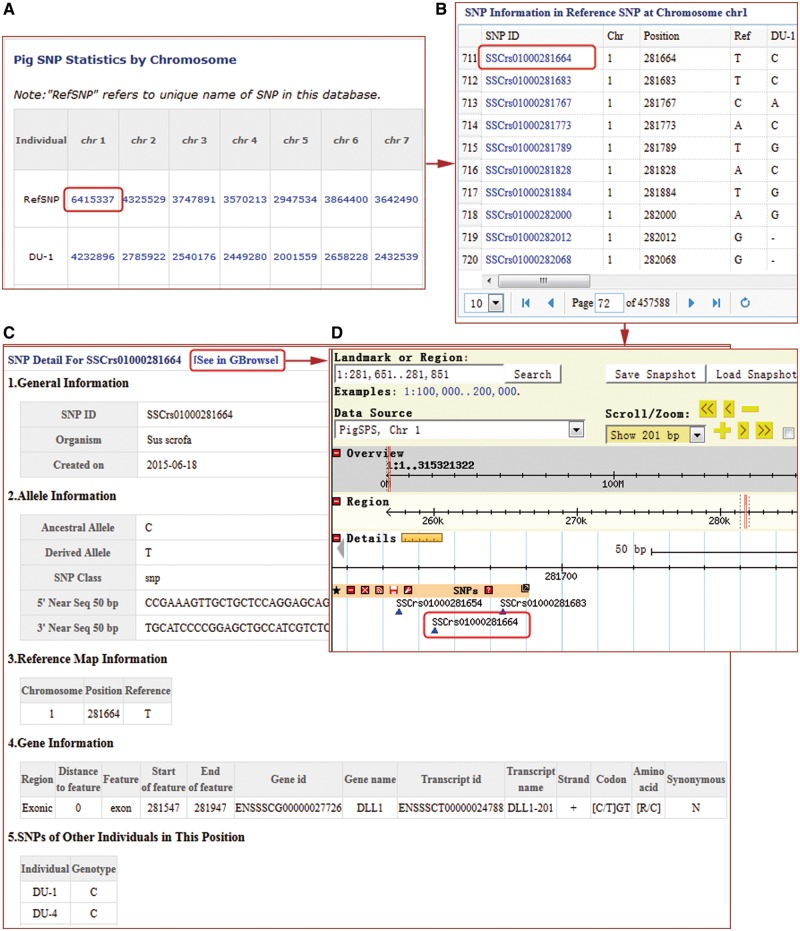
SNP search results windows in the PigVar database. (**A**) High-quality non-redundant SNP list for each sample. (**B**) SNP list for one chromosome. (**C**) Text format of the search results for *SSCrs01000281664* SNPs. (**D**) GBrowse results for *SSCrs01000281664*.

For SNPs, users can browse non-redundant and individual sample SNPs, either as tables in text format (Figure 2A and B), or chromosome-based GBrowse (30). Tables contain dbSNP ID.

PigVar ID, sample information, chromosome position, ancestral and derived alleles, as well as 50 bp of flanking sequences if available (Figure 2C). In addition, we have provided annotations for SNPs in protein-coding and lincRNA genes (Figure 2C). In the GBrowse interface, users can obtain SNP density information in 10k windows, related protein-coding or lincRNA gene and transcript information (Figure 2D). SNPs located in genic regions, especially non-synonymous SNPs, are often believed to be having important biological functions warranting further functional assessment. PigVar provides search functionality for SNP hits in protein-coding genes and lincRNA genes, as well as SNPs that result in synonymous and non-synonymous coding changes. PigVar allows users to search for SNPs singly or as a group by assigning chromosome numbers plus start and end locations. PigVar also has functions allowing comparisons of genotypes between two or more individuals.

**Figure 3.**
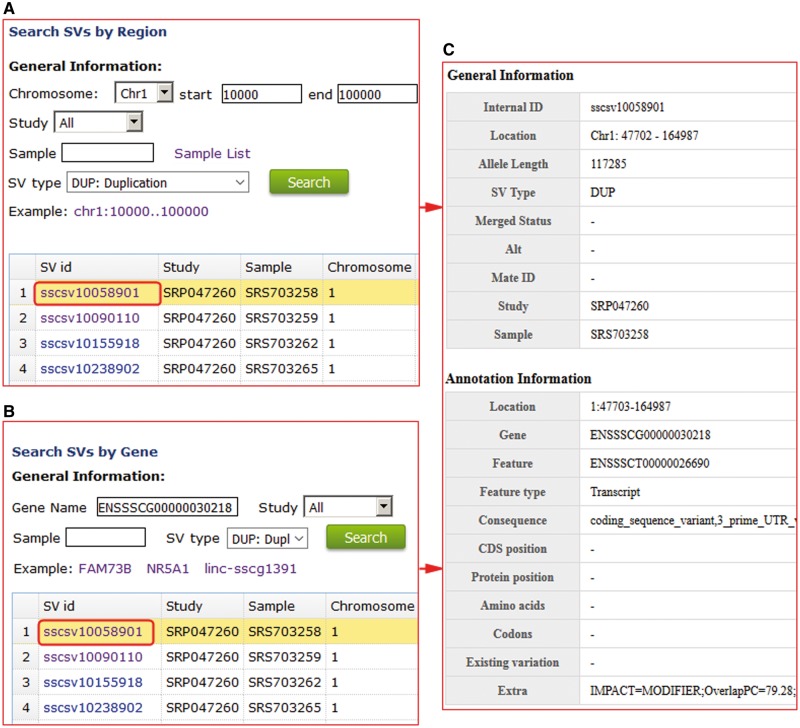
Structural variation search results in PigVar database. (**A**) Search by genomic region for structural variations (SVs). (**B**) Search by gene entry. (**C**) Text format of the search results for the gene ENSSSCG00000030218.

For structural variations, users can search the non-redundant and high-quality structural variations for every sample using genomic region or gene names (Figure 3A). We provide PigVar ID, sample location, chromosome position and SV types (Figure 3B). Users can search a given genomic position or genes to find samples that contain specific SVs, facilitating population genetic research in pigs.

**Figure 4.**
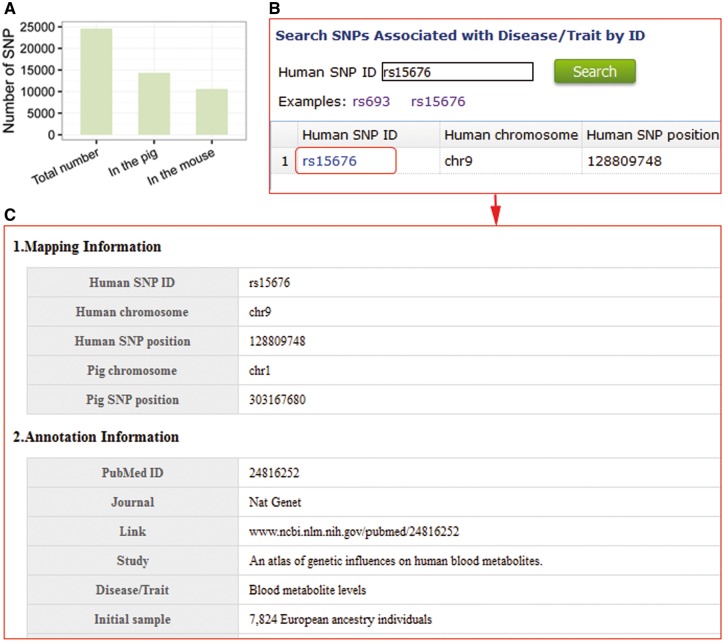
Pig is an excellent large-animal model for human diseases and traits. (**A**) Comparison of the number of SNPs in pigs and mouse that are homologous to SNPs in the NHGRI catalogue. (**B**) Search entry using a human SNP ID. (**C**) Detailed information of homologous porcine SNPs associated with human diseases genes and traits.

To model human diseases and traits, users can browse and search for porcine SNPs homologous to those associated with human diseases and traits using human SNP IDs (Figure 4B). SNP information and disease details, including SNP positions, annotation with genes and the details of the identification SNP associated with diseases, are provided in the database (Figure 4C).

**Figure 5.**
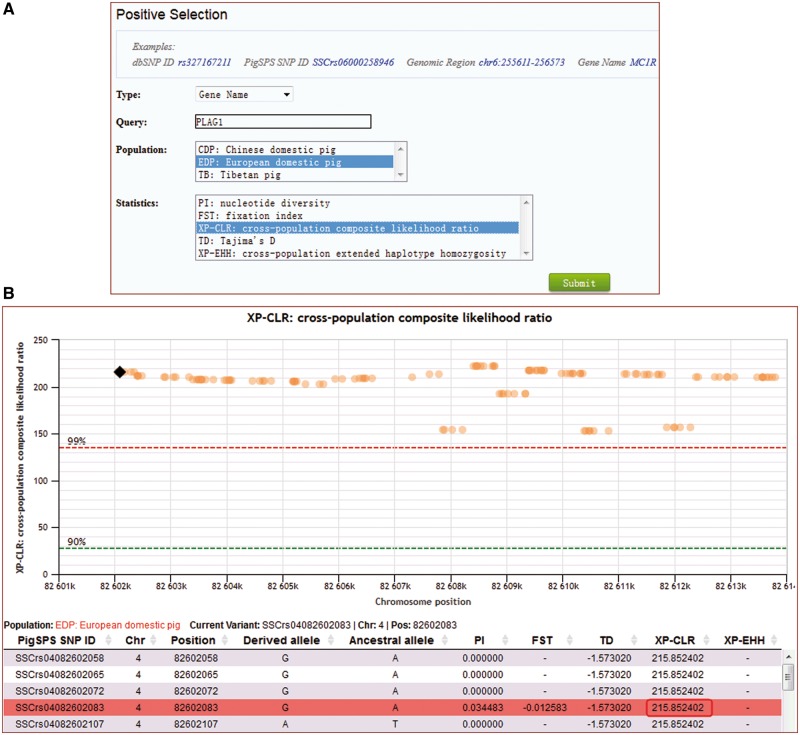
Search for signals of selection for the gene *PLAG1* by XP-CLR in European domestic pigs. (**A**) Caption of the search entry. (**B**) Selection signal results for *PLAG1*.

For searches of selection, users can select a specific pig population (Chinese domestic pigs, Tibetan pigs or European domestic pigs), and a specific location in one of four input formats, PigVar SNP ID, dbSNP ID, genomic region or gene name (Figure 5A). Genomic region can be a site or a region. Gene name supports RefGene name, Ensembl gene ID, and our lincRNA annotation IDs (26). Users can also choose any quantile in the calculated scores as the selection distribution cutoff (Figure 5B).

All SNP data called by samtools mpileup, GATK, phased SNPs, and the structure variations identified using LUMPY can be downloaded from the database.

## Discussion

PigVar is the first database that brings together SNPs called from 287 whole genome sequenced pigs and relatives, their selection scores, as well as structural variations from 211 pigs. Previous studies (7, 8, 13, 14, 20, 21) employed differing strategies to identify SNPs and SVs, while PigVar offers a unified SNP calling criteria via GATK (24, 25). Our approach identified a total of 64.6 million high-quality SNPs from 280 pig samples, and 36.8 million SNPs from 7 outgroup individuals. We also used a highly-accurate software, LUMPY (27), to identify 7 236 813 SVs in 211 pigs. Due to the high average sequencing depth, SNPs and SVs calling criteria, and sample distribution, our non-redundant variation data provides an extensive resource for examining genomic variation in pigs. SNPs are excellent markers for population genetic studies; therefore, we used the SNP data set to calculate signatures of selection in Chinese, Tibetan and European pigs. This resource should help researchers to efficiently identify loci that were putatively positively selected and allow further studies to assess the mechanisms behind the artificial or natural selections driving these changes. Users can download individual sample variation lists from our database to identify selection signatures for each breed. We also provide an annotation of the variations in protein-coding and long intergenic non-coding genes, which should contribute to our understanding of the causal mutations in pig evolution. Previous studies gave indications that many phenotypic changes are caused by structural variations or amino acid changes (7, 31). Thus, users of PigVar can combine SNP data, SVs data, annotation of variations, and positive selection signatures to identify candidate causal mutations.

Pigs are gaining increasing consideration as a model for medical research owing to the similarity of their metabolic characteristics, cardiovascular systems and organ size to those of humans (32), especially the miniature pig breeds such as the Bamaxiang pigs and Diannan small ear pigs. In this database, we provide SNP and SV data for these miniature pig breeds, which should contribute to research on new treatments for human diseases.

To ensure the long time efficiency of this database, we will regularly update the PigVar database as new pig whole-genome re-sequencing data by our own research group or from publicly available resources becomes available. PigVar will also undergo continuous improvements, for instance to enable downloading of intermediate processing files and submitting whole-genome sequence data or SNP list directly to our database by researchers. Additional statistical genetic parameters, such as CMS (33), iHS (34) and recombination maps, will also be added to the database, as well as selection scores for each breed.

In conclusion, PigVar represents the first comprehensive genomic pig database containing 64.6 million SNPs from 280 pigs, 36.8 million SNPs from their outgroup species, accurate structural variations in 211 pigs, as well as positive selection analysis. PigVar’s user friendly functionalities should enable researchers to obtain detailed information on specific SNPs, including location within protein-coding or lincRNA genes, synonymous or non-synonymous substitutions, ancestral or derived state, plus their associated positive selection scores. PigVar should accelerate medical research using pigs as a model, and enhance genomic studies including pig breeding.

## Supplementary data

Supplementary data are available at *Database* Online.

## Supplementary Material

Supplementary Data
